# Correlation versus Causation? Pharmacovigilance of the Analgesic Flupirtine Exemplifies the Need for Refined Spontaneous ADR Reporting

**DOI:** 10.1371/journal.pone.0025221

**Published:** 2011-10-11

**Authors:** Nora Anderson, Juergen Borlak

**Affiliations:** 1 Centre for Pharmacology and Toxicology, Hannover Medical School, Hannover, Germany; 2 Department of Molecular Medicine and Medical Biotechnology, Fraunhofer Institute of Toxicology and Experimental Medicine, Hannover, Germany; Bremen Institute of Preventive Research and Social Medicine, Germany

## Abstract

Annually, adverse drug reactions result in more than 2,000,000 hospitalizations and rank among the top 10 causes of death in the United States. Consequently, there is a need to continuously monitor and to improve the safety assessment of marketed drugs. Nonetheless, pharmacovigilance practice frequently lacks causality assessment. Here, we report the case of flupirtine, a centrally acting non-opioid analgesic. We re-evaluated the plausibility and causality of 226 unselected, spontaneously reported hepatobiliary adverse drug reactions according to the adapted Bradford-Hill criteria, CIOMS score and WHO-UMC scales. Thorough re-evaluation showed that only about 20% of the reported cases were probable or likely for flupirtine treatment, suggesting an incidence of flupirtine-related liver injury of 1∶ 100,000 when estimated prescription data are considered, or 0.8 in 10,000 on the basis of all 226 reported adverse drug reactions. Neither daily or cumulative dose nor duration of treatment correlated with markers of liver injury. In the majority of cases (151/226), an average of 3 co-medications with drugs known for their liver liability was observed that may well be causative for adverse drug reactions, but were reported under a suspected flupirtine ADR. Our study highlights the need to improve the quality and standards of ADR reporting. This should be done with utmost care taking into account contributing factors such as concomitant medications including over-the-counter drugs, the medical history and current health conditions, in order to avoid unjustified flagging and drug warnings that may erroneously cause uncertainty among healthcare professionals and patients, and may eventually lead to unjustified safety signals of useful drugs with a reasonable risk to benefit ratio.

## Introduction

Despite the vigorous and extensive safety testing during the drug development process rare adverse drug reactions (ADRs) of new medicinal products can not be detected at the time of market introduction. Consequently, ADRs are a leading cause for market removal of drugs [1]–[4] with drug-induced toxicities ranking among the top 10 causes of death in the US to result in health care costs of $30 billion US Dollars annually [5], [6]. Here, drug-induced liver injury (DILI) is the most frequent ADR and accounts for more than 50% of all cases of acute liver failure in the United States today [7].

As drug approvals are based on studies in 3,000–6,000 patients or less ADRs occurring in about 1 in 10000 patients cannot be detected in development programs. In addition, clinical study populations are inevitably pre-selected by study protocol-defined in- and exclusion criteria and are therefore not representative for the entire patient population exposed after market introduction. Therefore, prevention of ADRs remains the challenge of post-authorisation safety surveillance and it was estimated that approximately 600,000 subjects (1% of the population) exposed for one year would be required in order to reliably detect rare ADRs [8].

To overcome the safety gap between clinical studies and marketed products, pharmacovigilance aims to monitor, detect, understand and prevent ADRs (Table 1). Furthermore and as part of the FDA Post Marketing Drug Risk Assessment (PMDRA) program, pharmaceutical companies are requested to maintain a post-marketing surveillance system to provide Periodic Safety Update Reports (PSUR). Overall, national and international institutions gather a tremendous amount of data that necessitates data base entry and management, standardization procedures, statistical strategies and further processing and evaluation of the data. While pharmacovigilance databases can be used for the detection of early signals and may deliver evidence-based safety information for regulatory decision making [9], critical examination, along with careful interpretation and causality assessment of ADR data is required to eventually improve currently established drug safety concepts.

**Table 1:** Aims of post-marketing drug safety (pharmacovigilance) information reporting and management (adapted from Bate A et al., 2008 [28]).

*Aims of pharmacovigilance*	
***Signal detection***	*To detect previously unknown adverse drug effects after drug approval*
***Discovery of subgroup at risk***	*To evaluate risks in subpopulations (based on age, sex, main diagnosis and disease)*
***Estimation of ADR incidence***	*To estimate all of adverse drug reactions in relation to additional information such as sales volume (provided by manufacturers)*
***Support of risk-benefit analyses***	*To estimate the risk of potentially toxic drugs and compare it to their beneficial therapeutic potential*
***Discovery of potential drug-drug interactions***	*To detect previously unknown drug-drug interactions and to estimate incidences of known drug-drug interactions*
***Hypotheses generation for off-target drug effects***	*To propose mechanisms of ADRs based on prospective or retrospective analysis of clinical data*
***Risk management strategies***	*Strategies and recommendations for an identification of individuals at risk to improve safety of drugs*

Here, we address some challenges and limitations of current pharmacovigilance practice and processes. We choose the analgesic flupirtine as an example and evaluated the spontaneous ADR reports that led in Germany to a signal of hepatotoxicity. Using this drug as an example we wish to highlight the potential pitfalls and limitations in drug safety evaluation of ADR reports and to stimulate a discussion on improved ADR reporting and assessment.

An evaluation of spontaneous reports on suspected adverse reactions kindly provided by the German health authority (BfArM) and the Drug Commission of the German Medical Association is presented to probe for the evidence of adverse hepatobiliary events reported in association with the administration of the analgesic flupirtine. Specifically, flupirtine is a central non-opioid analgesic with muscle-relaxing properties, which is classified as a first in class Selective Neuronal Potassium (KCNQ) Channel Opener (SNEPCO) [10], [11].

Since its approval in Germany in 1984 flupirtine serves as an alternative for non-steroidal anti-inflammatory drugs (NSAIDs) and cyclooxygenase-2 (COX-2) inhibitors primarily for the treatment of pain associated with degenerative changes of the musculoskeletal system and conditions associated with painful muscle tension or spasms (e.g. lumbalgia). Flupirtine provides analgesia without cardiac, renal and gastrointestinal adverse effects, including bleeding complications, and others that limit the therapeutic use of NSAIDs in pain management. Moreover, flupirtine separates from typical side-effects profile of opioids such as respiratory depression potential or constipation.

Prescriptions numbers for flupirtine continue to rise with approximately 17 millions defined daily doses in 2006, an increase of about 40% compared to the previous year [12].

In 2007 the Drug Commission of the German Medical Association (AkdÄ) released a notification on the hepatotoxic potential of flupirtine that was based on ADRs reported over a 16-year period (1992–2007). Concern was expressed that the incidence of flupirtine-related liver injury may have been underestimated. Thus, the signal “hepatobiliary ADR/liver toxicity”, which was detected only by number of reports but not by plausibility test of causality evaluation of spontaneous reports, was communicated as a signal for a possible general hepatotoxicity of flupirtine [13].

In the following we present the results of a standardized Medical Dictionary for Regulatory Activities (MedDRA) query and the evaluation of individual spontaneous reports in order to verify whether or not the signal “hepatobiliary ADR/liver toxicity”, is substantiated and reproducible in the majority of individual ADR cases. Our thorough analysis of all reported cases yielded no evidence for flupirtine to qualify as liver toxin.

## Methods

A standardized MedDRA query for reports of hepatobiliary adverse events in association with flupirtine treatment was carried out using the German health authority (BfArM) data base for suspected adverse drug reactions in February 2009.

The query retrieved a total of 229 reports, of which 3 cases were excluded since they were reported twice. Cases were assessed using the WHO-UMC causality assessment system (www.who-umc.org) and the adapted Bradford-Hill criteria [14], [15]. The Council for International Organizations of Medical Sciences/Roussel Uclaf Causality Assessment Method scale (CIOMS score), as discussed by Teschke et al., 2008 [16], was used as reference scale. Statistical correlations were based on linear regression analysis using the Statistica software, Version 8.0 (Statsoft Inc., Tulsa, USA). Data sets used for statistical evaluations are provided as supplementary material (Table S1).

Based on prescription numbers for the years 1992–2008 that amounted to defined daily doses (DDD) of 155.2 millions with a median drug intake of 56 days (estimated from n = 175 of 226 cases, where information on duration of drug intake was available), the incidence of flupirtine-related hepatobiliary adverse events was estimated to be about 0.8 in 10,000 patients. This is considered to be a very rare frequency of hepatobiliary ADRs that was calculated the following way:

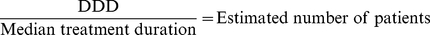


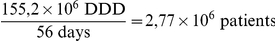

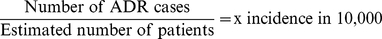


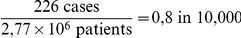

## Results

### Spontaneously reported hepatobiliary reactions related to flupirtine exposure

In the years 1992–2008 a total of 226 spontaneous individual reports of hepatobiliary adverse events related to flupirtine exposure were notified to and recorded by the German Federal Institute for Drugs and Medical Devices (BfArM). The majority of the reports were provided by healthcare professionals; others were reported by the marketing authorisation holder of flupirtine, clinical study directors and patients. Some basic demographic information on sex and age of the cohort is provided in Table 2.

**Table 2:** Sex and age distribution in 226 cases of spontaneously reported suspected ADRs to the German Federal Institute for Drugs and Medical Devices.

	No. of cases	[%]
**Sex of patients**		
Total cases	226	100
Female patients	171	76
Male patients	52	23
No information	3	1
**Age distribution**		
<40 years	3	1
40–60 years	126	56
>60 years	80	35
No information	18	8

**Figure 1:**
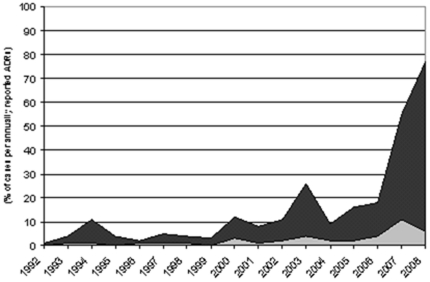
Annually reported cases of flupirtine induced liver injury. Annually spontaneously hepatobiliary adverse events reported for flupirtine (black) and proportion of cases rated to be ‘highly probable’ or ‘probable’ according to the CIOMS score (grey).

About 76% of 226 patients were female (for 3 patients no gender information was available), and the age of most patients was between 40 to 60 years (56%) or older (35%). The sex distribution agrees well with registered prescriptions, i.e. 33.3% for male and 66.7% for female patients for the years 1992–2008 according to IMS Health Incorporated data. Six cases with a fatal outcome were reported in association with flupirtine administration. The median daily dose of flupirtine was reported with 300 mg and the median duration of exposure was 56 days. Numbers of annually reported cases are provided in Figure 1.

### Correlation versus causation

To evaluate the causality of drug exposure for a reported suspected ADR, the data have to be probed for their statistical association and for the validity, reliability and consistency of the reported evidence [17]–[19]. The theoretical basis for an assessment of a causal relationship evaluation between two factors was established by Sir Austin Bradford-Hill in 1965 [15]. These criteria include strength, consistency, specificity, temporality, biological gradient, plausibility, coherence, experimental evidence and analogy. Since then, the Bradford-Hill criteria have been widely used in epidemiology and may be, with some limitations, applied to pharmacovigilance and pharmacoepidemiology as well [14]. A summary of the adapted Bradford-Hill criteria applied to the pharmacovigilance data base obtained from the German Federal Institute for Drugs and Medical Devices (BfArM) for flupirtine is given in Table 3. The findings in Table 3 will be discussed in conjunction with an evaluation based on the CIOMS and WHO-UMC causality assessment system described below (see also supplementary Table S4 for a classification of the severity of the ADR cases according to who-umc.org).

**Table 3:** Causality assessment according to the adapted Bradford-Hill criteria.

*Criteria*	*Findings*	*Evidence*
***Strength of association***	*No statistical association between dose/duration & exposure of flupirtine and response in laboratory parameters ALT, AST, bilirubin (see * *Figure 1* *) or alkaline phosphatase (AP)*	
		*ADR incidence below 1% * [*23]*, [29]–[35**] * – Long-term controlled open tolerability study for 12 months (n = 244 patients): according to laboratory values no effects on liver function (DD 100–600 mg) * [*26*].
***Consistency of association***	***Clinical studies:*** * At least 6 published clinical trials at the Pubmed database; * ***Literature reports do not provide evidence for hepatobiliary side effects***	*various reports on the pharmacokinetics and metabolism of flupirtine * [*36]*–[38**] * – e.g. 1 report on a putative protective mechanism on mitochondria (liver) * [*39*] *.*
		*- e.g. various reports on anti-apoptotic and neuroprotective activities * [*40]*–[44**]
		*e.g. 1 report on use in patients with impaired liver function * [*35*]
***Dose-Response Relationship***	*No statistically significant correlation between the daily and cumulative dose and the clinical chemistry parameters ALT, AST and bilirubin*	*[for analysis see supplementary data]*
		*Number of cases in * ***direct*** * temporal relationship (<14 days:) 30/176 cases, thereof are 9/176 cases with time relation ≤2 days, 12/176 cases with time relation >2 and ≤7 days and 9/176 cases with time relation >7 and <14 days*
***Temporal Relationship***	*No information regarding the time to onset of ADR in 50/226 cases*	*Number of cases with a * ***possible*** * temporal relationship: 17/176 cases with time relation 14–30 days*
		*Number of cases with * ***no direct*** * temporal relationship (>30 and <365 days): 121/176 cases [for analysis see supplementary data]*
		*Number of cases with an * ***unlikely*** * temporal relationship: 8/176 cases with a time relation >365 days*
	*Number of cases where cessation of adverse events (AE) after discontinuation of treatment was reported: 164/22*	*98/164 positive dechallenge*
		*66/164 unclear dechallenge*
		*10/66 very slow decrease of laboratory parameters*
		*56/66 decrease of laboratory parameters after discontinuation of flupirtine and at least one other potential hepatotoxic drug*
	*Number of cases where cessation of AE after discontinuation of treatment was not reported 62/226*	*6/62 could not be evaluated due to death of the patients*
		*49/62 no information available*
		*7/62 dechallenge did not resolve condition (causality unlikely)*
***Coherence/Specificity***	*At total of 23/226 cases with reported re-exposure*	
	*A total of 14/23 with reported outcome*	*13/14 positive re-challenge result (causality assumed)*
		*1/14 negative re-challenge result (no causality assumed)*
	*A total of 167/226 cases with reported co-medications*	*151/167 cases where co-medication(s) could be responsible or contributing factor(s) for hepatobiliary ADRs*
		*51/167 cases where physician explicitly suspected co-medication to be causally involved in the reported ADR*
***Plausibility***	*Exclusion of other causes, such as infections, alcohol, disease-related causes, other drugs (see text and * *Table 4* *)*	

Summary of the analyses of 226 spontaneous suspected ADRs reports.

**Figure 2:**
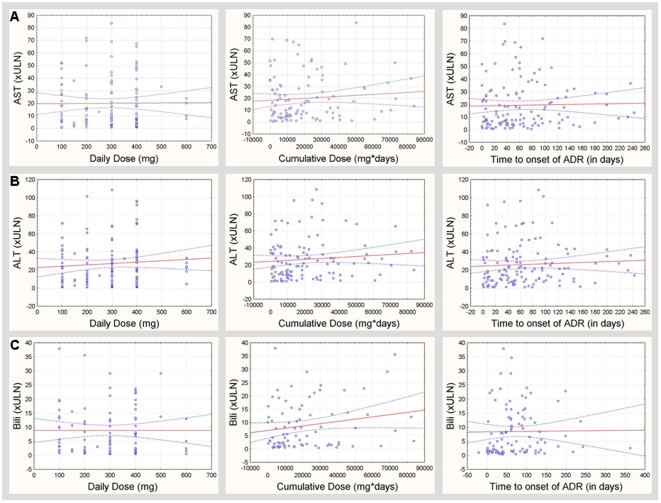
Results of liver function tests of spontaneously reported ADRs. Scatter plots of maximum AST (A), ALT (B), and bilirubin (C) levels (given in×ULN) in relation to the daily dose (left panel), cumulative doses (daily dose (mg)×duration of drug exposure (days), middle panel), and in relation to the time to onset (right panel) of the ADRs in cases of suspected hepatobiliary adverse events associated with flupirtine exposure.

Figure 2 displays scatter blots of the laboratory parameters aspartate aminotransferase (AST) (Figure 2A), alanine aminotransferase (ALT) (Figure 2B) and bilirubin (Figure 2C) (in × upper limit of normal (ULN)) in relation to daily dose of flupirtine, cumulative dose, as well as time to onset (TTO) of the suspected ADR. Statistical analyses did not evidence any significant relationship between the respective parameters (see Figure 2). Size of data sets and p-values are provided as supplementary material (Tables S2 and S3).

### Plausibility check based on history of drug intake (pharmacoanamnesis)

Step wise exclusion of alternative causes for the reported adverse drug events is part of an aetiology based approach for a causal assessment of ADRs, such as the “French approach” [20], [21]. Likewise, an application of the Bradford-Hill criteria for the plausibility of the causal relation between drug and event involves exclusion of alternative aetiologies affecting the hepatobiliary system, such as infections (viral, bacterial, parasites), metabolic diseases (e.g. non-alcoholic steatohepatitis), storage diseases (M. Wilson, haemochromatosis), auto-immune diseases, other systemic diseases (lupus erythematodes, chronic inflammatory bowels disease amongst others). Table 4 lists the information reported on exclusion of viral and autoimmune-related causes of hepatobiliary symptoms reported as suspected ADRs of flupirtine.

**Table 4:** Incidence of viral disease and cases with positive autoimmune titres in 226 cases of liver ADR linked to flupirtine treatment.

	No. of cases	[% of all 226 cases]
**Results for cases with autoimmune antibodies reported**	**50**	**22.1**
*Autoimmune antibodies positive*	*18*	*8.0*
*Autoimmune antibodies negative*	*32*	*14.2*
**Results unclear**	**2**	**0.9**
**No information**	**174**	**77.0**

	No. of cases	[% of all 226 cases]
**Results for cases with reported hepatitis serology**	**106**	**46.9**
Hepatitis A positive (total)	19	8.4
*Hepatitis A acute infection*	*3*	*1.3*
*Hepatitis A old infection*	*16*	*7.1*
*Hepatitis infection status unclear*	*4*	*1.8*
Hepatitis B positive	1	0.4
Hepatitis C positive	1	0.4

Information on exclusion of other hepatotropic viruses was provided in a few cases only. For 26 patients test results were reported for EBV (in 24 cases), CMV (13 cases) and VZV (5 cases). No cases with hepatitis E infection were reported.

There were 22 reported cases of alcohol abuse and 11 cases of diabetes mellitus that had been suspected as possible confounders for the observed liver toxicity during flupirtine therapy.

To evaluate the hepatic safety profile of flupirtine, co-medications needed to be assessed as well. Out of 167 cases (73.9% of all 226 cases) with reported co-medication 151 cases (90.4% of 167 cases) received co-medication that included one or more medicines labelled for ADRs affecting the hepatobiliary system (summary of product characteristics (SmPC) contained information on hepatobiliary ADRs).

The median number of additional co-medications with the potential for hepatobiliary ADRs was two with a large scatter (average: 2.8±3.1). In 51 out of 167 cases notified, the reporter had suggested the co-medications as a possible cause of the suspected adverse reaction. Table 5 provides an overview of the 10 most common co-medications in the ADR reports on flupirtine labelled for hepatobiliary ADRs. The 10 most common co-medications suspected for a causal/contributing relation to the reported ADRs with flupirtine are listed in Table 6.

**Table 5:** The 10 most common co-medications with a potential for hepatobiliary ADRs in n = 151 reported cases with co-medications possibly responsible or contributing factor(s) for hepatobiliary ADRs.

No.	Medication	No. of cases	% of 151 cases
1	Ibuprofen	19	12.6
2	ACE Inhibitors	19	12.6
3	Acetylsalicylic acid	17	11.3
4	Amitriptyline	17	11.3
5	Coxibs	16	10.6
6	Diclofenac	17	11.3
7	Tramadol	17	11.3
8	Statins	15	9.9
9	Metamizol	14	9.3
10	Estradiol	14	9.3

**Table 6:** The 10 most common co-medications of n = 51 cases reported by physicians as a possible cause of hepatic ADRs.

No.	Medication	No. of cases reported as suspected	No. of cases with this drug as com-medication	%
1	Amitriptyline	5	17	9.8
2	Coxibs (Rofecoxib, Lumiracoxib, Etoricoxib, Celecoxib)	5	16	9.8
3	Estradiol	4	14	7.8
4	Diclofenac	3	17	5.9
5	Doxepin	2	9	3.9
6	Fluvastatin	2	4	3.9
7	Gabapentin	2	5	3.9
8	Ibuprofen	2	19	3.9
9	Levothyroxine	2	10	3.9
10	Metamizol	2	14	3.9

In the following two case reports are briefly described where flupirtine was rated as either unlikely or certain for ADR.

#### Case report 1

While hospitalized, a 32-year old male patient diagnosed with Guillain Barre Syndrome had received flupirtine, ibuprofen, and metamizole as pain medication. Reported co-medications were: pregabalin, insulin, promethazine, enoxaparin and potassium citrate. After 3 days of flupirtine administration (600 mg/day) he displayed increased ALT (4×ULN) and AST levels (3×ULN). Treatment with flupirtine and pregabalin was discontinued. With a latency of 6 days he was re-exposed to pregabalin and responded with massively increases in ALT (28×ULN) and AST (24×ULN) levels. Drug-induced liver injury – induced by pregabalin was rated certain.

As described above the 226 cases also included cases, where co-medications rather than concomitant diseases (hepatitis) were more likely to be responsible for the hepatobiliary ADR. Specifically, in case no. 1 re-challenge with pregabalin led to a massive increase of serum transaminases. This case can therefore be rated as unlikely for flupirtine exposure to cause DILI. In contrast, in case no. 2 (see below) and due a positive re-challenge with flupirtine the reported ADR was rated to be “certain” (WHO/UMC system) or “possible” (CIOMS score). The difference in the score between both causality assessment systems is related to the lack of clinical information for case no. 2 (e.g. other than viral causes), which have a higher weight in the CIOMS score.

#### Case report 2

**Figure 3:**
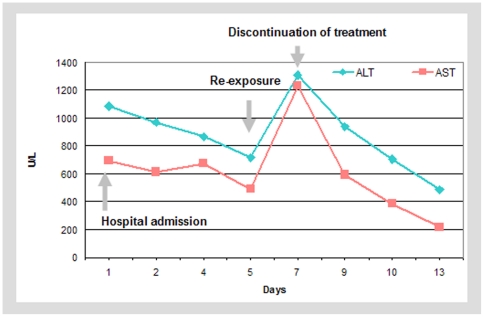
Flupirtine hyper-sensitivity of a 53-year-old female patient. Laboratory results of a 53-year-old female patient after exposure and re-exposure to flupirtine. She experienced icterus with markedly increased serum transaminase levels. Upon discontinuation transaminase levels decreased. On day 6 of hospital admission re-exposure to an unknown dose of flupirtine resulted in a recurrent increase of laboratory values.

A 53-year-old female patient was admitted to the hospital and diagnosed with jaundice. Viral and autoimmune hepatitis were excluded. The patient had taken flupirtine on demand (unknown daily dose) for the treatment of pain related to cervical spine syndrome. The patient displayed increased transaminases (ALT: 31.0×ULN, AST: 19.8×ULN). Co-medications were estradiol+norethisterone and zolpidem on demand. After discontinuation of flupirtine the lab parameters decreased. Re-exposure (dose unknown) on day 5 of admission resulted in a recurrent increase of laboratory parameters (see Figure 3 below).

A systematic review of the 226 spontaneous individual reports of hepatobiliary ADRs associated with flupirtine intake defined 57 cases with elective liver biopsies. However, information on liver biopsies for 49 cases could be retrieved only and the findings are summarised in Table 7 (note, for each case several diagnoses are listed). Essentially for 23 cases inflammatory hepatitis was confirmed by histopathology while histopathological features consistent with toxic liver damage were reported for 36 cases. Furthermore, in 29 out of the 36 cases (i.e. >80%) with toxic liver damage, co-medications with other drugs known to cause hepatobiliary ADRs were reported as well and in n = 6 cases autoimmune-related changes were excluded, but in 18 of the 226 patients positive titres for auto antibodies were noted.

**Table 7:** Histopathological findings in liver biopsies taken from n = 49 patients with reported hepatobiliary ADRs (multiple histological findings were listed for one patient).

Diagnosis	Cases confirmed	Cases excluded	Cases possible	Cases with no information
***Toxic liver damage***	*36*	*0*	*0*	*13*
***Fibrotic changes***	*15*	*1*	*0*	*33*
***Necrotic changes***	*30*	*0*	*0*	*19*
***Cirrhotic changes***	*1*	*3*	*0*	*45*
***Steatotic changes***	*3*	*5*	*0*	*41*
***Cholestatic changes***	*10*	*7*	*0*	*32*
***Inflammatory reactions***	*23*	*0*	*0*	*26*
***Autoimmune related changes***	*0*	*6*	*5*	*38*
***Virus hepatitis***	*0*	*11*	*1*	*37*
***Alcohol-related changes***	*0*	*2*	*0*	*47*
***Morbus Wilson (Cu)***	*0*	*4*	*0*	*45*
***Haemochromatose (Fe)***	*1*	*12*	*0*	*36*
***Changes of the bilary duct system***	*2*	*9*	*0*	*38*

Neither anti-nuclear antibodies (ANAs) nor anti-mitochondrial antibodies (AMAs) are sufficiently specific for the diagnosis of an autoimmune liver disease. Positive titres for these are also found in other conditions, such as collagenosis, rheumatoid arthritis, as well as primary biliary cirrhosis, which could however be excluded, based on the available histopathology findings. Furthermore, in one case the liver specific protein (LSP) was determined, but reported as negative.

### Findings from re-challenge of individual patients

To further probe for a possible causal relationship of hepatobiliary ADRs patients may be re-challenged with the suspected drug. However, this constitutes significant risks for the patient and should only be attempted if the drug is required to treat a serious disease and no alternative medication is available. In the present study and mainly due to medically unsupervised re-exposure by the patients themselves, information on liver transaminases was obtained. Out of 226 cases with hepatobiliary ADRs, re-challenge was reported in 15 cases with information on the outcome available for n = 14 cases (6.2% of 226 cases). In n = 13 cases (5.8% of 226 cases) re-challenge resulted in a re-occurrence or worsening of the symptoms, while in 1 case no increase of transaminases was observed.

Apart from these well defined cases the following assumption may be justified. In 59 out of 226 cases no information on co-medication was available but at least for 32 of these 59 cases (or 14.2% of 226 cases), an amelioration of symptoms upon treatment discontinuation (positive de-challenge) was observed. The lack of additional information does not permit firm conclusions to be drawn from these cases which are confounded by the likely use of additional but unreported pain medications. Thus, the positive de-challenge may simple result from the complete withdrawal of all drugs used in the pain management of these patients.

Generally, a positive re-challenge results in an increase of transaminases after re-exposure with a suspected drug and this indicates a likely causal relationship, provided other potential causes can be excluded with sufficient certainty (see case report 2).

### Causality assessment by use of the CIOMS and WHO-UMC scales

The results of the evaluation based on the CIOMS scale and the WHO-UMC causality assessment system are provided in Table 8.

**Table 8:** Evaluation of 226 cases with suspected hepatobiliary ADRs reported for flupirtine obtained with the CIOMS score and the WHO-UMC causality assessment system.

	CIOMS		WHO	
	[No. of cases]	[% of total cases]	[No. of cases]	[% of total cases]
highly probable	7	3.1	14	6.2
probable	33	14.6	19	8.4
possible	78	34.5	124	54.9
unlikely	71	31.4	33	14.6
excluded	37	16.4	36	15.9

*WHO-UMC causality assessment system categories were assigned to CIOMS score categories as follows: certain = highly probable+probable; probable/likely = probable, (highly probable); possible and possible/unclassified = possible; unlikely = unlikely, unclassified and unclassifiable = excluded.*

After subtracting 36 reports witch lacked essential information, 190 cases were analyzed for possible aetiologies according to WHO-UMC causality assessment system [8], [9], [14], [18], [19]. Causality assessment rated 14 cases to be certain, 19 cases to be probable, 124 cases to be possibly and 33 cases to be unlikely associated with flupirtine treatment. The result for each case was compared with an evaluation according to the CIOMS scale. Both classifications agreed by about 55%. 176 cases of reported suspected ADRs rated by the WHO-UMC causality assessment system did not provide a certain (93% of 190 included cases) and 157 cases (83%) not a probable link to flupirtine intake. Thus, the majority of cases was not rated likely (highly probable) or probably related to flupirtine exposure. As shown in Table 8, the number of cases differed between the two causality assessment systems. Different outcome between various causality assessment scales has been observed by others (as recently reported by Garcia-Cortes et al., 2008 [22]). We view the CIOMS scale particularly useful as it addresses more vigorously hepatobiliary ADRs.

In 6 of the reported ADR cases (2.6%) with possible/probable causality (according to WHO/UMS assessment) to flupirtine exposure the symptoms associated with the event were consistent with allergic/pseudo-allergic drug reactions (acute symptoms shortly after flupirtine administration, earlier reported exposure, fever, rash, nausea, gastrointestinal symptoms or respiratory distress), which are largely independent from dose [23]. The lack of the dose-relationship and the close temporal relation to flupirtine exposure classifies a total of 19 cases (not infectious, no potential hepatotoxic co-medication, no other co-medication, no autoimmune antibodies, no other causes, no alcohol, time to onset of ADR<90 days) as possible/probable idiosyncratic type B reactions.

As discussed above we infer monotherapy with flupirtine in 59 out of 226 cases since no other information is available. The lack of additional information does not permit firm conclusions to be drawn about the cases since the suggested association between flupirtine and hepatobiliary events might be confounded by various unreported hepatotoxic pain medications. Notably, NSAIDs were the most common co-medication (59 of 226 cases = 26.1%) in hepatobiliary ADR reports for flupirtine. Mostly, NSAIDs were prescribed to the patients for the same indication as flupirtine as part of a combination therapy of the underlying pain condition. As shown in Tables 5 and 6, co-medications consisted of numerous drugs with a potential to cause hepatobiliary ADRs or even DILI, with one patient receiving up to 25 drugs. Furthermore, as shown in Figures S1 and S2 of the supplementary data, the number of drugs given and the severity of liver damage appeared to be linked in patients suffering from a variety of pain conditions to either the use NSAIDs or other drugs known or suspected to be hepatotoxic.

## Discussion

Pharmacovigilance aims at an understanding and the prevention of adverse drug effects to enhance patient safety in relation to the use of medicines. There is a need for reliable and balanced information for the effective assessment of the risk-benefit profile of medicines.

Based on these premises we report the case of flupirtine where limitations of causality assessment of ADR filings resulted in distorted signal detection for DILI. A thorough analysis provided little evidence for flupirtine to be reasonably suspected as a candidate drug with a remarkable liability for causing hepatotobiliary adverse events. Rather, management of pain frequently requires complex co-medication with drugs well known for their hepatobiliary adverse event profile, such as NSAIDs. Our data analysis establishes that pain relief with flupirtine does not impose a higher risk for hepatobiliary ADRs or DILI based on the incidence that was estimated to <1 in 10,000 as compared to NSAIDs. For instance, a recent study estimated the incidence of severe hepatobiliary ADRs requiring hospitalization in patients receiving the NSAID diclofenac to 23 in 100,000 patients [24], [25]. Based on prescription numbers for the years 1992–2008 that amounted to defined daily doses (DDD) of 155.2 millions with a median drug intake of 56 days (estimated from n = 175 of 226 cases, where information on duration of drug intake was available), the incidence of flupirtine-related hepatobiliary adverse events was estimated to be about 0.8 in 10,000 patients.

When confounded cases that were rated to be of unlikely causality or that could definitely be excluded would not be considered in these estimates, the “true” (i.e. less confounded) incidence would be even lower than the estimate given above.

The low incidence of hepatobiliary ADRs is also supported by findings in various clinical studies, in which flupirtine was administered over long term periods (up to 12 months) under well controlled clinical conditions without signs of liver toxicity [26]; [Clinical trial (1988): Investigation of the efficacy and tolerance of the analgesic flupirtine in patients who regularly need analgesics for long periods (uncontrolled open multicentre study), not published]; Owner: MEDA Pharma GmbH & Co. KG, Bad Homburg, Germany] and there are basically no published case reports in regards to flupirtine hepatobiliary ADRs, despite a more than 20 year-use of this drug. Note that the incidence of hepatobiliary ADRs reported for common NSAIDs is 3 to 23 per 100,000 patients and therefore higher than that of flupirtine [24].

Based on the pharmacovigilance data base entries, it is highly probable that the complex co-medication with drugs labelled with hepatobiliary ADR profiles may well be responsible for the majority of the hepatobiliary ADRs reported for flupirtine.

We did, however, identify rare cases of pseudo allergic/idiosyncratic reactions that might occur with an incidence of less then 1 in 100,000. This estimate is based on 19 reported cases and >2.7 million patients that were treated with an estimated average duration of 56 days as discussed above. In November 2007 the Drug Commission of the German Medical Association (AkdÄ) released a warning for flupirtine that was followed by an apparent increase in spontaneously reported cases in 2008. This apparent increase in ADR reporting, however, may be more reasonably explained by the considerable increase in prescription numbers that occurred during that time, and perhaps also may be related to an increase in awareness of possible hepatobiliary ADRs in response to the warning by the medical authority, a challenging phenomenon in drug safety assessment. Despite an increase in crude numbers of ADR reporting in the years 2007 (kind of over-reporting) and 2008 of more than 7-fold compared to the average ADR reporting in earlier years, the number of cases, which were rated ‘likely’ or ‘probably’ remained largely unchanged as depicted in Figure 1.

Although the release of a warning raises public attention and might have either a beneficial or confounding impact in signal detection, it should not be taken as a substantial evidence for a suspected adverse drug reaction without a thorough causality assessment of each spontaneously reported case.

Impaired hepatic function in patients with primary liver diseases or alcohol-induced liver injury (i.e. severe liver cirrhosis) are labelled contra-indications for flupirtine, since these conditions may predispose to CNS-related adverse reactions to flupirtine, such as ataxia and exaggeration of symptoms of hepatic encephalopathy.

Evaluation of the 226 ADRs identified a majority of cases that were confounded by medical history, intercurrent diseases involving cases of hepatic steatosis (see Table 7), abuse of alcohol and/or drugs, and other co-medications which themselves carry the potential of hepatobiliary ADRs. The strong association between the number of co-medications and the reported cases of hepatobiliary ADRs during flupirtine therapy are important for the causality assessment. Unfortunately, the number of cases of mono therapy with flupirtine could not be determined precisely, simply because no reliable information was available. We therefore assumed 59 cases on mono-therapy with flupirtine. For 32 cases an improvement was reported after drug withdrawal that would represent 14.2% out of 226 cases or 54% out of 59 cases. Nonetheless, it is likely that these patients had received additional medications (note for 167 cases co-medication was reported and in the majority of cases (90.4% of 167 cases) co-medication included one or more medicines labelled for ADRs affecting the hepatobiliary system). Indeed, co-medication particularly with drugs known to have hepatobiliary side effect profile such as NSAIDs and antidepressants may have been either the primary cause or at least significant contributors to liver injury also in cases attributed to flupirtine. Pain management often requires complex co-medications, which means that causality with regards to hepatobiliary safety signals for a given drug needs to be assessed in conjunction with all other parts of the treatment regimen. Particularly elderly patients have a reduced hepatic metabolism and renal clearance, as well as a reduced distribution volume. An adjustment of their therapy in regard to dose and choice of drugs has to take their increased ADR risk profile into account.

Our data show a direct link between the number of drugs given and the severity of liver impairment suggesting that a combination of COX-2 inhibitors or NSAIDs itself or with flupirtine may significantly aggravate and/or increase the incidence of hepatobiliary ADRs. This is in line with an analysis of 54,583 reports of suspected adverse drug reactions from the French Pharmacovigilance database that suggested a six- to sevenfold higher risk for severe ADRs (hepatic injury and acute renal failure) when two NSAIDs or more were administered at the same time [27]. In light of the well known and broad spectrum of adverse drug reactions related to NSAIDs the high number of cases with NSAID as co-medications is not surprising. One reason for the high number of co-medications in some ADR reports is the possibility to obtain drugs over the counter without prescription as part of a “self medication plan”. The poor awareness regarding the risk profile of OTC drugs has been the subject of several warnings by the regulatory authorities. In hepatobiliary ADR reports on flupirtine drug-drug interactions, for instance with NSAIDs, appear to have been a major contributing factor.

### Current pitfalls in pharmacovigilance

**Figure 4:**
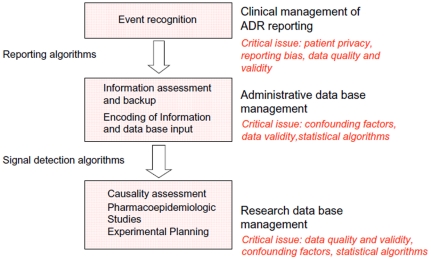
Reporting scheme and critical issues in the management of suspected ADRs.

Pharmacovigilance is of utmost importance to provide information on drug exposure and safety/tolerability in a large and inhomogeneous unselected patient population. Lack of relevant information regarding aetiologies, medical history, and co-medications can lead to misconceptions and distorted signals of ADRs as summarised in Figure 4.

While data mining is of great importance for pharmacovigilance there are unmet needs in the development of algorithms for the reliable detection of ADR signals. Unfortunately currently used data mining algorithms are not vigorously validated and do not objectively screen spontaneous reported data. As of today neither supervised nor unsupervised mathematical models are available that can be used reliable for the causality assessment of spontaneous reported ADRs. Furthermore, current data mining algorithms are not designed for hypothesis generation nor can they be used to develop a balanced perspective that can be reasonable validated. There is a need to improve the quality of data collected and to develop an automised procedure for the careful assessment and collation of spontaneous ADR reports that will permit the effective detection of hitherto unknown adverse drug reactions – a process known as “signal detection”. The quality and practical usefulness of spontaneous pharmacovigilance reporting systems is dependent on the completeness of the reports and the validity, reliability and overall plausibility of the data. The development of novel algorithms / interrogation procedures used in pharmacovigilance will help to ascertain the plausibility and causality of reported ADRs. Indeed, the current causality evaluation systems (WHO and CIOMS) do not discriminate between genuine drug-related adverse events and drug-drug interactions that result in ADRs, but such interplay of different risk factors need to be considered for an improved discriminatory power and overall risk benefit assessment.

### The road ahead

Our study exemplifies the gap between currently implemented spontaneous ADR reporting systems and plausibility of causality assessment. It is of paramount importance to develop simple strategies in order to improve the content-quality of spontaneously reported pharmacovigilance information based on patient factors and a thorough drug history.

Vigorous and thorough causality assessment, as suggested in the present study, has to be implemented into the national and local pharmacovigilance routine and the daily clinical practise. It should be noted that information on causality is required by regulatory authorities and causality assessment is performed by the marketing authorization holder; however this information frequently remains incomplete and poorly validated and sometimes may be even lost in the current pharmacovigilance process. Although a vast amount of literature regarding various methods of data base management, statistical analysis and signal detection algorithms is available, it is still the input-quality and completeness of information that matters (i.e. garbage in – garbage out!) and there appears to be still major room for improvements.

## Supporting Information

Figure S1 **Alanin and Aspartate transaminase activities in patients treated with flupirtine NSAIDs and other drugs.** ALT (A) and AST (B) (in×ULN) in relation to the number of drugs with potential hepatobiliary ADRs in cases, where patients had received NSAIDs as co-medications. Data are given as means ± standard error of the mean. Abbreviations: FL (flupirtine alone), HT (potential hepatotoxin, i.e. 1 to 4 additional drugs or above).
(DOC)

Figure S2 **Alanin and Aspartate transaminase activities in patients treated with flupirtine or other drugs considered as potential hepatotoxins.** ALT (A) and AST (B) (in×ULN) in relation to the number of drugs with potential hepatobiliary ADRs in cases. Data are given as means ± standard error of the mean. Abbreviations: FL (flupirtine alone), HT (potential hepatotoxin, i.e. 1 to 6 additional drugs or above). Elevated clinical chemistry parameter may simple reflect the dose of the total number of hepatotoxins given to the patient. Figure S1 depicts the influence of the number of co-medications with a potential for hepatobiliary ADRs on the ALT and AST levels of patients which received NSAIDs. Figure S2 depicts the influence of the number of co-medications with other potentially hepatotoxic drugs in patients with available ALT and AST activities.
(DOC)

Table S1 **Clinical chemistry and pathology of 226 cases of flupirtine induced drug liver injury.** Data sets available for the analyses and evaluation of 226 cases of spontaneously suspected ADRs related to Flupirtine intake and reported to the German Federal Institute for Drugs and Medical Devices.
(DOC)

Table S2 **Statistical analysis of laboratory parameters of 226 cases of flupirtine induced drug liver injury.** Statistical analysis of laboratory parameters of 226 reported cases in regards to duration of drug exposure (days) and time to onset (as defined by the reporting health professional) of ADR (in days).
(DOC)

Table S3 **Statistical analysis of laboratory parameters of 226 cases of flupirtine induced drug liver injury.** Statistical evaluation of laboratory parameters obtained from 226 serious ADRs in relation to the daily (in mg) or cumulative dose (mg×days).
(DOC)

Table S4 **Severity of 226 ADRs cases.** (WHO definition, see www.who-umc.org).
(DOC)
