# Supplementary material for: Correlation versus Causation? Pharmacovigilance of the Analgesic Flupirtine Exemplifies the Need for Refined Spontaneous ADR Reporting
Source: PLoS One. 2011 Oct 11;6(10):e25221. doi: 10.1371/journal.pone.0025221 (PMC3191146; doi:10.1371/journal.pone.0025221)
Supplement: Figure S1 — Alanin and Aspartate transaminase activities in patients treated with flupirtine NSAIDs and other drugs. ALT (A) and AST (B) (in×ULN) in relation to the number of drugs with potential hepatobiliary ADRs in cases, where patients had received NSAIDs as co-medications. Data are given as means ± standard error of the mean. Abbreviations: FL (flupirtine alone), HT (potential hepatotoxin, i.e. 1 to 4 additional drugs or above). (DOC) [file pone.0025221.s001.doc]

**Supplementary Figure S1**

ALT (A) and AST (B) (in x ULN) in relation to the number of drugs with potential hepatobiliary ADRs in cases, where patients had received NSAIDs as co-medications. Data are given as means ± standard error of the mean. Abbreviations: FL (flupirtine without drugs with potential hepatobiliary ADRs as co-medication), group + X (number of additional drugs with potential hepatobiliary ADRs), HT (potential hepatotoxin), n = X (number of ADR cases).
