# Supplementary material for: Correlation versus Causation? Pharmacovigilance of the Analgesic Flupirtine Exemplifies the Need for Refined Spontaneous ADR Reporting
Source: PLoS One. 2011 Oct 11;6(10):e25221. doi: 10.1371/journal.pone.0025221 (PMC3191146; doi:10.1371/journal.pone.0025221)
Supplement: Figure S2 — Alanin and Aspartate transaminase activities in patients treated with flupirtine or other drugs considered as potential hepatotoxins. ALT (A) and AST (B) (in×ULN) in relation to the number of drugs with potential hepatobiliary ADRs in cases. Data are given as means ± standard error of the mean. Abbreviations: FL (flupirtine alone), HT (potential hepatotoxin, i.e. 1 to 6 additional drugs or above). Elevated clinical chemistry parameter may simple reflect the dose of the total number of hepatotoxins given to the patient. Figure S1 depicts the influence of the number of co-medications with a potential for hepatobiliary ADRs on the ALT and AST levels of patients which received NSAIDs. Figure S2 depicts the influence of the number of co-medications with other potentially hepatotoxic drugs in patients with available ALT and AST activities. (DOC) [file pone.0025221.s002.doc]

**Supplementary Figure S2**

**Figure S2:** ALT (A) and AST (B) (in x ULN) in relation to the number of drugs with potential hepatobiliary ADRs in cases. Data are given as means ± standard error of the mean. Abbreviations: FL (flupirtine without drugs with potential hepatobiliary ADRs as co-medication), group + X (number of additional drugs with potential hepatobiliary ADRs), HT (potential hepatotoxin), n = X (number of ADR cases).

Elevated clinical chemistry parameter may simple reflect the dose of the total number of hepatotoxins given to the patient. Figure 2 depicts the influence of the number of co-medications with a potential for hepatobiliary ADRs on the ALT and AST levels of patients which received NSAIDs. Figure 3 depicts the influence of the number of co-medications with a potential for hepatobiliary ADRs in patient with available ALT and AST levels.
