# Supplementary material for: Correlation versus Causation? Pharmacovigilance of the Analgesic Flupirtine Exemplifies the Need for Refined Spontaneous ADR Reporting
Source: PLoS One. 2011 Oct 11;6(10):e25221. doi: 10.1371/journal.pone.0025221 (PMC3191146; doi:10.1371/journal.pone.0025221)
Supplement: Table S1 — Clinical chemistry and pathology of 226 cases of flupirtine induced drug liver injury. Data sets available for the analyses and evaluation of 226 cases of spontaneously suspected ADRs related to Flupirtine intake and reported to the German Federal Institute for Drugs and Medical Devices. (DOC) [file pone.0025221.s003.doc]

**Supplementary Table S1**

Data sets available for the analyses and evaluation of 226 cases of spontaneously suspected ADRs related to Flupirtine intake and reported to the German Federal Institute for Drugs and Medical Devices.

| **Information** | **Available data (no. of cases)** | **Available data (% of total cases)** |
| --- | --- | --- |
| Sex | 223 | 98.7% |
| Age | 208 | 92.0% |
| Dose (in mg) | 167 | 73.9% |
| Duration of drug intake (days) | 175 | 77.4% |
| Duration of ADR | 132 | 58.4% |
| ALT (xULN) | 159 | 70.4% |
| AST (xULN) | 157 | 69.5% |
| Bilirubin (xULN) | 121 | 53.5% |
| AP (xULN) | 89 | 39.4% |
| Gammaglutamyl transferase (GGT) (xULN) | 148 | 65.5% |
| Lactate dehydrogenase (LDH) (xULN) | 51 | 22.6% |
| Information on indication for flupirtine | 194 | 85.8% |
| Cases of flupirtine mono therapy (no comedication reported) | 59 | 26.1% |
| Co-medications | 167 | 73.9% |
| Biopsies | 57 | 25.2% |
| Information on histopathological evaluation | 49 | 21.7% |
| Information on hepatitis serology | 106 | 46.9% |
| Information on auto antibodies | 52 | 23.0% |
