# Supplementary material for: Correlation versus Causation? Pharmacovigilance of the Analgesic Flupirtine Exemplifies the Need for Refined Spontaneous ADR Reporting
Source: PLoS One. 2011 Oct 11;6(10):e25221. doi: 10.1371/journal.pone.0025221 (PMC3191146; doi:10.1371/journal.pone.0025221)
Supplement: Table S2 — Statistical analysis of laboratory parameters of 226 cases of flupirtine induced drug liver injury. Statistical analysis of laboratory parameters of 226 reported cases in regards to duration of drug exposure (days) and time to onset (as defined by the reporting health professional) of ADR (in days). (DOC) [file pone.0025221.s004.doc]

**Supplementary Table S2**

Statistical analysis of laboratory parameters of 226 reported cases in regards to duration of drug exposure (days) and time to onset (as defined by the reporting health professional) of ADR (in days)

|  |  | Included data sets/total data sets | Linear Correlation Coefficent [r] | Correlation (p-value) | Coefficient of Determination [r2] |
| --- | --- | --- | --- | --- | --- |
| ALT (xULN) | *Duration of dug intake (days)* | 133/138 | 0.0075 | 0.9316 | 0.0001 |
|  | *Time to onset of ADR (days)* | 132/140 | 0.0563 | 0.5231 | 0.0032 |
| AST (xULN) | *Duration of dug intake (days)* | 123/128 | 0.0525 | 0.5645 | 0.0028 |
|  | *Time to onset of ADR (days)* | 123/131 | 0.0259 | 0.7761 | 0.0007 |
| Bilirubin (xULN) | *Duration of dug intake (days)* | 100/103 | 0.1435 | 0.1565 | 0.0206 |
|  | *Time to onset of ADR (days)* | 99/104 | 0.0118 | 0.908 | 0.0010 |
| AP (xULN) | *Duration of dug intake (days)* | 73/76 | -0.0310 | 0.7958 | 0.0010 |
|  | *Time to onset of ADR (days)* | 70/76 | 0.0041 | 0.9734 | 0.0000 |

Abbreviations: times upper the limit of normal (xULN)
