# Supplementary material for: Correlation versus Causation? Pharmacovigilance of the Analgesic Flupirtine Exemplifies the Need for Refined Spontaneous ADR Reporting
Source: PLoS One. 2011 Oct 11;6(10):e25221. doi: 10.1371/journal.pone.0025221 (PMC3191146; doi:10.1371/journal.pone.0025221)
Supplement: Table S3 — Statistical analysis of laboratory parameters of 226 cases of flupirtine induced drug liver injury. Statistical evaluation of laboratory parameters obtained from 226 serious ADRs in relation to the daily (in mg) or cumulative dose (mg×days). (DOC) [file pone.0025221.s005.doc]

**Supplementary Table S3**

Statistical evaluation of laboratory parameters obtained from 226 serious ADRs in relation to the daily (in mg) or cumulative dose (mg x days)

|  |  | Included data sets/total data sets | Linear Correlation Coefficent [r] | Correlation (p-value) | Coefficient of Determination [r2] |
| --- | --- | --- | --- | --- | --- |
| ALT (xULN) | *Dose [mg]* | 127/129 | 0.0805 | 0.3681 | 0.0065 |
|  | *Cumulative dose (dose [mg] x intake [days])* | 116/119 | 0.0955 | 0.3123 | 0.0091 |
| AST (xULN) | *Dose [mg]* | 117/119 | 0.008 | 0.9319 | 0.0001 |
|  | *Cumulative dose (dose [mg] x intake [days])* | 104/109 | 0.0878 | 0.3731 | 0.0077 |
| Bilirubin (xULN) | *Dose [mg]* | 89/92 | -0.0002 | 0.9987 | 0.0000 |
|  | *Cumulative dose (dose [mg] x intake [days])* | 81/84 | 0.1939 | 0.0828 | 0.0376 |
| AP (xULN) | *Dose [mg]* | 74/77 | 0.0087 | 0.9415 | 0.0001 |
|  | *Cumulative dose (dose [mg] x intake [days])* | 65/68 | -0.0313 | 0.8045 | 0.0010 |

Abbreviations: times upper the limit of normal (xULN)
