# Supplementary material for: Correlation versus Causation? Pharmacovigilance of the Analgesic Flupirtine Exemplifies the Need for Refined Spontaneous ADR Reporting
Source: PLoS One. 2011 Oct 11;6(10):e25221. doi: 10.1371/journal.pone.0025221 (PMC3191146; doi:10.1371/journal.pone.0025221)
Supplement: Table S4 — Severity of 226 ADRs cases. (WHO definition, see www.who-umc.org). (DOC) [file pone.0025221.s006.doc]

**Supplementary Table S4**

Severity of 226 ADRs cases (WHO definition, see [www.who-umc.org](http://www.who-umc.org/))

| **Severity of ADR cases** | **No. of cases** | **% of total** |
| --- | --- | --- |
| no information | 12 | **5.3** |
| non serious | 23 | **10.2** |
| serious (D) | 5 | **2.2** |
| serious (DA) | 4 | **1.8** |
| serious (H) | 80 | **35.4** |
| serious (L) | 24 | **10.6** |
| serious (other medical important conditions | 52 | **23.0** |
| serious (prolonged hospitalisation) | 26 | **11.5** |
|  | 226 | **100.0** |

*Abbreviations: D (death), DA (disabeling), H (hospitalization), L (life threatening)*
